# Temporal structure of brain oscillations predicts learned nocebo responses to pain

**DOI:** 10.1038/s41598-021-89368-0

**Published:** 2021-05-07

**Authors:** Mia A. Thomaidou, Joseph S. Blythe, Simon J. Houtman, Dieuwke S. Veldhuijzen, Antoinette I. M. van Laarhoven, Andrea W. M. Evers

**Affiliations:** 1 Faculty of Social and Behavioral Sciences, Leiden University, Wassenaarseweg 52, 2333 AK Leiden, The Netherlands; 2 Leiden Institute for Brain and Cognition, 2333 ZA Leiden, The Netherlands; 3 Department of Integrative Neurophysiology, Center for Neurogenomics and Cognitive Research, Amsterdam Neuroscience, VU University Amsterdam, 1081 HV Amsterdam, The Netherlands; 4 Department of Psychiatry, Leiden University Medical Center, 2333 ZA Leiden, The Netherlands; 5 Medical Delta Healthy Society, Leiden University, Technical University Delft, Erasmus University Rotterdam, Rotterdam, The Netherlands

**Keywords:** Biomarkers, Neuroscience, Cognitive neuroscience, Sensory processing, Somatosensory system

## Abstract

This study aimed to identify electrophysiological correlates of nocebo-augmented pain. Nocebo hyperalgesia (i.e., increases in perceived pain resulting from negative expectations) has been found to impact how healthy and patient populations experience pain and is a phenomenon that could be better understood in terms of its neurophysiological underpinnings. In this study, nocebo hyperalgesia was induced in 36 healthy participants through classical conditioning and negative suggestions. Electroencephalography was recorded during rest (pre- and post-acquisition) and during pain stimulation (baseline, acquisition, evocation) First, participants received baseline high thermal pain stimulations. During nocebo acquisition, participants learned to associate an inert gel applied to their forearm with administered high pain stimuli, relative to moderate intensity control stimuli administered without gel. During evocation, all stimuli were accompanied by moderate pain, to measure nocebo responses to the inert gel. Pre- to post-acquisition beta-band alterations in long-range temporal correlations (LRTC) were negatively associated with nocebo magnitudes. Individuals with strong resting LRTC showed larger nocebo responses than those with weaker LRTC. Nocebo acquisition trials showed reduced alpha power. Alpha power was higher while LRTC were lower during nocebo-augmented pain, compared to baseline. These findings support nocebo learning theories and highlight a role of nocebo-induced cognitive processing.

## Introduction

The experience of pain varies widely between and within individuals and can be shaped by cognitive processes such as learning. Nocebo hyperalgesia, a worsening in perceived pain attributed to negative expectations, demonstrates that learning can be detrimental for the experience of pain^1–3^. Memories and negative expectations may directly impact pain processing^4,5^, yet it remains unclear which specific processes are involved in cognitive pain reappraisal and how negative expectations may shape physiological characteristics of pain.

Electroencephalography (EEG) can be used to identify physiological markers of phenomena that include cognitive components^6,7^ such as nocebo effects. EEG has been used in cognitive and pain research and has largely focused on spectral characteristics of brain oscillations, with evidence indicating that expectations^8,9^ and cognitive pain regulation^10,11^ are reflected through alterations in the alpha and beta power bands. Concurrently, EEG research has shown that gamma oscillations are involved in associative learning^12^ and encoding of ongoing pain^13^. Alpha and gamma oscillations may also act in synergy during the cognitive stages of nociceptive processing^14^. Previous research^15^ showed an overall effect of nocebo acquisition on some EEG parameters form pre- to post-acquisition. How specific EEG measures within frequency bands relate to pain and cognitive processing under hyperalgesic conditions remains unclear.

Electrophysiological research into nocebo effects has been scarce and has mainly focused on the power spectrum of oscillations^15–19^. However, in order to more precisely pinpoint cognitive processes involved in nocebo, it may be valuable to utilize sophisticated EEG biomarkers such as Detrended Fluctuation Analysis (DFA), a component that quantifies long-range temporal correlations (LRTC) between oscillating groups of neurons and determines how oscillation amplitudes change over time^20^. Higher LRTC generally indicate higher complexity of neural activity and have accordingly been shown to play a role in cognitive processes such as attention and cognitive reappraisal^20–22^. Decreases in LRTC of oscillations have been found in schizophrenia^23^ and Alzheimer's disease^24^, with both disorders being characterized by cognitive deficiencies. Moreover, strong LRTC of beta and gamma oscillations have been associated with poor sustained attention performance^25^. Despite its evident and intricate relationship to cognitive processing, complexity of brain activity has never been tested under nocebo hyperalgesic conditions.

As described, we based this study on earlier findings relating to changes in (resting-state) oscillatory power in the alpha band. Additionally, we aimed to explore nocebo correlates relating specifically to LRTC of brain oscillations during active pain states throughout the experiment. We expected that the magnitude of induced nocebo hyperalgesia would be positively correlated to pre- to post-acquisition LRTC alterations in the alpha band, while we expected the opposite relationship in the beta and gamma bands. Furthermore, we expected that the experience of control versus nocebo trials during the acquisition and evocation phases would be characterized by divergent EEG biomarker values. Additionally, we expected that the experience of nocebo-augmented pain and baseline high-pain stimulations would be characterized by divergent EEG biomarker values. Finally, we explored the relationship between pain-related psychological characteristics and measures of EEG, to investigate the relationship between nocebo effects and psychological constructs that are directly related to pain, such as catastrophizing and fear of pain, as well as cognitive intrusions on pain, variables that may be relevant to participants’ pain responses^15^ in this nocebo experiment.

## Materials and methods

### Participants

Participants of either sex were enrolled in this study. The required sample size for the primary analysis was calculated based on a previous nocebo study^18^ that induced nocebo hyperalgesia on thermal pain by use of conditioning, in an MEG paradigm. This study was used merely as an indicator of an appropriate sample size for this comparable study, in lack of a more fitting study to base a power analysis on. Tu et al.^18^ found that a decrease in alpha band connectivity predicted the magnitude of conditioned nocebo hyperalgesia (*r* = 0.46, *p* = 0.04). The power analysis was conducted in G*power 3.1^26^ for our primary hypothesis. Alpha error probability was set at α = 0.05, and desired power was set at 0.80. With *r* of 0.46, the sample size indicated was 36 participants. A replacement protocol was used for excluded participants.

Inclusion criteria were: age between 18 and 35 years, a good understanding of the English language, and (corrected to) normal vision and hearing. Exclusion criteria were pregnancy or breastfeeding, any pain on the day of testing, having recent injuries on the arms, painful health conditions experienced in the past 6 months, ever having experienced chronic medical or psychiatric conditions, and having consumed psychotropic or analgesic medication, recreational drugs, or more than 3 units of alcohol, in the 24 h prior to the study appointment. Testing of included participants was discontinued in the case that they would be determined to have too high of a pain threshold (i.e., when thermode maximum temperatures were not sufficient to induce at least moderate pain) or when they would not reliably report a difference (a mean of at least 1.5 on the NRS) between the administered temperatures for control and nocebo trials in the acquisition phase. Participants were recruited through the online website Sona (Sona Systems, Tallinn, Estonia). Study participation involved a 3-h recording session at a laboratory of the Faculty of Social and Behavioral Sciences of Leiden University, the Netherlands. All participants provided written informed consent prior to participation. After completing the experiment, all participants were reimbursed by either study credits or cash. The study was approved by the Leiden University Psychology Research Ethics Committee (CEP19-1031/532; all methods and procedures were performed in accordance with the relevant guidelines and regulations) and pre-registered on ClinicalTrials.gov (NCT04199858, 16/12/2019; planned analyses of frequency biomarkers were not conducted due to the scope of this paper).

### Experimental design

**Figure 1:**
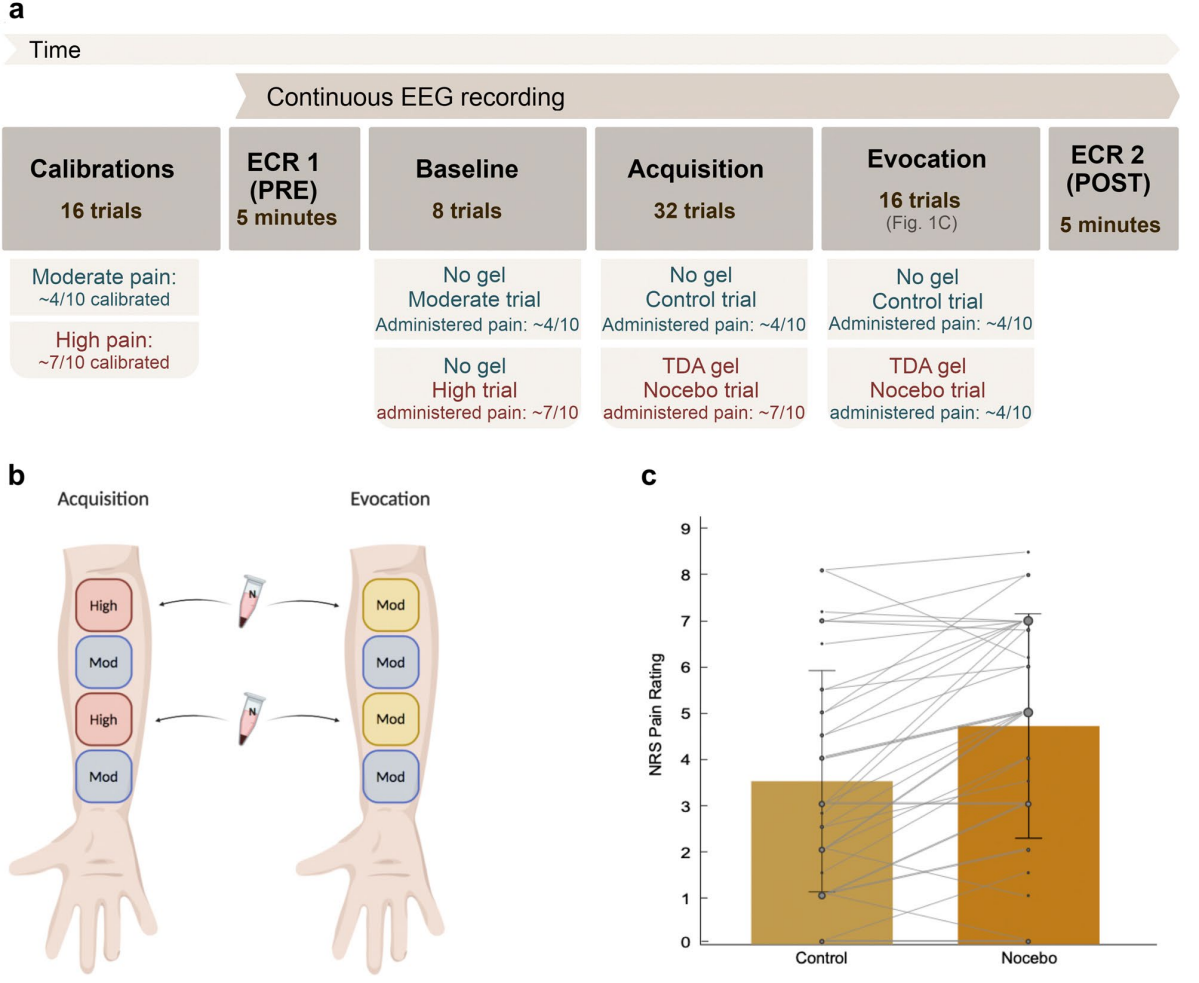
Experimental protocol and induced nocebo effect. (**a**) All participants underwent pain calibrations before continuous EEG measurements. At the start of the recording, participants completed a first (PRE) resting-state, received (baseline) moderate and high pain stimulations, and underwent nocebo acquisition via conditioning and verbal suggestions, nocebo evocation, and a second (POST) resting-state. Blue and red fonts indicate the lower and higher pain conditions, respectively. *ECR* Eyes-Closed Rest, *PRE* Pre-acquisition, *POST* Post-acquisition, *TDA* Trans-Dermal Aspartate (sham hyperalgesic gel). Approximate pain in (**a**) represents the moderate and high pain stimulations administered during acquisition and evocation, while (**c**) represents reported pain during the moderate pain stimulations of the evocation phase. (**b**) Application sites where either no gel or sham hyperalgesic gel was applied. Starting from the most distal patch on the right volar forearm, either no gel was applied on control sites or the sham hyperalgesic gel “TDA” was applied on the nocebo sites. During nocebo acquisition, moderate thermal pain was administered on control sites and high pain was administered on nocebo sites. During evocation, pain stimulations were administered at moderate intensity. (**c**) Manipulation-check results showing the pain ratings for the first nocebo and the first control trials of the evocation phase for all participants (*n* = 36). Error bars depict standard deviation.

This study utilized a within-subjects design. All participants underwent (1) a calibration phase, (2) a baseline phase, and a nocebo phase comprising (3) nocebo acquisition and (4) nocebo evocation (Fig. 1a). During the first phase, calibrations for warmth and pain perception were conducted. During the baseline phase, moderate- and high-pain stimuli were administered. During nocebo acquisition, a conditioning procedure took place, in which associations were learned between the nocebo treatment and higher pain. Participants were conditioned to associate a sham pain-increasing gel with high (increased) pain stimulations, and no gel (control) with moderate-pain stimulations. During nocebo evocation, these learned associations were tested.

### Thermal pain application

Thermal pain stimuli were delivered to the volar forearm using a Thermal Sensory Analyzer with a 3 × 3 cm ATS thermode probe (TSA-II; Medoc Advanced Medical Systems, Ramat Yishai, Israel). During the calibrations and baseline phases, both arms were used for pain stimulations. During the nocebo phase, only the right arm was used (Fig. 1b). Throughout the experiment, pain intensities were rated on a numeric rating scale (NRS) ranging from 0 (no pain) to 10 (worst pain imaginable in this context).

#### Sensory and pain thresholds

Before the start of the experimental phases, warmth and pain threshold levels were tested for each participant, heat stimuli were applied and participants were asked to indicate the first moment at which they perceived warmth and pain. After a practice trial for each, the average of 3 warmth detection values and 3 heat pain detection values determined the thresholds for warmth and pain, respectively. This method follows published standardized procedures^27^.

#### Pain calibration protocol

Pain calibrations were conducted in order to determine the temperatures that would induce moderate and high pain during baseline and nocebo phases. The calibrations were individually tailored, based on the NRS ratings of 16 heat stimuli of varying intensities. Throughout the experiment, each pain stimulus was initiated from a 32 °C baseline, increased to a target temperature, and presented for 10 s at plateau. The ramp up and return rates were 8 °C per second. During calibrations the inter-stimulus interval (ISI) was 5 s, during which NRS pain ratings were given. Median temperatures rated as NRS 3 to 5 were used to induce moderate pain and median temperatures rated as NRS 6 to 8 were used to induce high pain.

#### Baseline, acquisition, and evocation phases

During baseline, 2 moderate and 6 high pain trials were administered on both arms, with an ISI of 5 s. During acquisition, 16 nocebo and 16 control stimuli were administered in alternating order. During evocation, 8 nocebo and 8 control stimuli were administered in alternating order. During nocebo acquisition and evocation, the ISI was 10 s. In all phases the thermode was moved to a more proximal site on the arm after each pain trial, in order to avoid habituation or sensitization to heat-pain.

### Nocebo manipulation

A commercial moisturizing gel that was given the name “Trans-Dermal Aspartate” or “TDA” was used as the nocebo treatment in the procedure; participants were told it was a capsaicin gel used on the skin for research purposes only. Half of the participants received the gel from a blue jar and the other half from a brown jar, both featuring sham pharmaceutical labels. Negative suggestions were used to create expectations regarding the pain enhancing effects of the gel. Participants were told that the gel is a capsaicin-based gel that is known for its pain-increasing properties. Participants’ arms were marked with medical tape to create four 3 × 3 cm thermode-placement sites on both arms. Prior to the start of the acquisition phase, the gel was rubbed into the two nocebo sites (the first and third most proximal sites on the right arm). We opted for stimuli to be administered on the same side for all participants, for more clarity when assessing any potential laterality in the results. Messages displayed on a computer screen via E-Prime 2.0 (Psychology Software Tools, Pittsburgh, PA, USA) indicated whether a trial was on a gel site or on a control site. The messages read “Trans-Dermal Aspartate, pain-increasing capsaicin, gel form” or “Control trial, no gel”.

During nocebo acquisition, the nocebo gel was paired to surreptitiously increased pain stimulations during nocebo trials, while moderate pain was delivered during control trials. During nocebo evocation, all pain stimuli during both nocebo and control trials were applied at moderate intensity, to study whether evoked conditioned responses were elicited. Increased pain reports for a nocebo trial as compared to its preceding control trial in this phase indicated nocebo hyperalgesia.

### EEG materials

EEG recordings were conducted using the ActiveTwo BioSemi (Amsterdam, the Netherlands) electrode system from 32 scalp electrodes. As reference electrodes, BioSemi replaces the ground electrodes that are used in conventional systems with two additional electrodes. The Common Mode Sense active electrode and Driven Right Leg passive electrode form a feedback loop, which drives the average potential of the participant as close as possible to the reference voltage of the analog-to-digital converter, thus rendering them references. Data was acquired at a sampling rate of 1024 Hz, band-passed filtered online during acquisition from 0.1 to 100 Hz (with a 100 Hz low-pass and 0.01 Hz high-pass hardware filter). Electrodes were placed on the scalp according to the international 10–20 system and where possible, electrode impedances were kept below 20 kOhm.

### Questionnaires

Three questionnaires were used to measure baseline differences in psychological characteristics. The questionnaires were completed by participants prior to their lab visit. Total scores were used for the following questionnaires: The Pain Catastrophizing Scale (PCS; Sullivan et al.^28^), the Fear of Pain Questionnaire (FPQ-III; McNeil and Rainwater^29^), and the Experience of Cognitive intrusions on Pain scale (ECIP; Attridge et al.^30^). At the end of the experiment, participants also completed an exit questionnaire containing manipulation check questions, assessing, for example, whether participants understood the instructions. All questionnaires, as well as a debriefing form, were displayed via web-based survey software (Qualtrics, Provo, Utah, USA).

### Experimental procedure

Before the day of testing, participants completed a brief online screening as well as the psychological questionnaires. On the day of the testing session, participants received further information about the procedures and provided written informed consent. Then, participants completed a brief screening for inclusion and were provided with information about the EEG and the (sham) pain-enhancing effects of the nocebo gel. EEG caps were then mounted, electrolyte gel was applied (SignaGel, Parker laboratories Inc., Fairfield, New Jersey, USA) and the scalp electrodes were placed. Warmth and pain threshold levels were then tested and individual pain stimuli were calibrated. Thereafter, continuous EEG recording started and the baseline phase was completed. Participants then completed a 5-min resting-state recording with their eyes closed. Then, participants underwent nocebo acquisition and evocation. Subsequently, participants completed a second 5-min resting-state recording. After the end of the experiment, participants completed the exit questionnaire. Finally, a debriefing was conducted and participants were reimbursed for their participation.

### Data handling

Analyses of behavioral data were performed for descriptive purposes and to confirm that a significant nocebo effect was induced. Next, specific hypotheses were tested, starting with resting-state EEG data. Our primary hypothesis was that there would be pre- to post-acquisition decreases in LRTC in the alpha band, given the role of alpha oscillations in pain processing as well as previous findings regarding the role of oscillatory complexity in cognitive functions. We then examined whether direct links could be observed between nocebo-induced changes in resting-state brain activity (pre- to post-acquisition) and the magnitude of induced nocebo hyperalgesia, with the aim to identify resting-state correlates of nocebo hyperalgesia. We then examined EEG parameters during the experience of pain stimulations. We first asked whether the experience of control and nocebo trials during the acquisition and evocation phases would be characterized by divergent EEG biomarker values. We then focused on potential differences in brain activity during the experience of high pain at baseline and the experience of heightened pain under nocebo hyperalgesic conditions (i.e., when lower pain stimulation is perceived as high pain, during nocebo evocation). Finally, we explored the correlation between pain-related psychological questionnaires and measures of EEG.

#### Nocebo manipulation check

The magnitude of reported nocebo hyperalgesia was measured within-subjects, and was defined as the difference in pain ratings for the first nocebo trial compared to the first control trial, during evocation. The first evocation trials were selected to answer the manipulation-check question of whether significant nocebo hyperalgesia was induced, as previous studies indicate the effect to be clearest in those trials^31,32^.

#### Behavioral data handling

Behavioral data were analyzed by use of SPSS 23.0 (IBM Corp., Armonk, NY, USA). The threshold for significance was set at *P* < 0.05 and partial eta-squared ($\eta_{p}^{2}$) was computed as a measure of effect size, with $\eta_{p}^{2}$ of 0.01 considered small, 0.06 considered medium, and 0.14 considered a large effect size^33,34^. To conduct repeated measures analysis of variance (ANOVA), the assumptions of normality and homogeneity of variances were checked.

#### Computation of EEG biomarkers

Spectral and temporal biomarkers were computed for all EEG recordings within three canonical frequency bands: alpha (8–13 Hz), beta (13–30 Hz) and gamma (30–45 Hz). To quantify local neural dynamics associated with resting-state brain activity and pain responses in our nocebo paradigm, spectral power was computed for all EEG electrodes using the *Welch* method implemented in MATLAB. Relative power was computed as the relative contribution of power within a narrow band to the integrated power within the range 1–45 Hz. To investigate whether temporal structure of the EEG changed at rest and during pain responses in our nocebo paradigm, the amplitude envelope was extracted using the Hilbert transform and DFA was computed to quantify LRTC of neuronal oscillations^20–22^. DFA quantifies the rate at which auto-correlations of amplitude modulations decay within a signal, with the power-law exponent ranging from 0.5 (uncorrelated) to 1.0 (strong auto-correlations). Signals were filtered using a FIR-filter with a Hamming window with a length corresponding to two *f*_1_ Hz cycles for a given frequency band [*f*_1_, *f*_2_]. To minimize artificial auto-correlations introduced by the FIR-filter, DFA was fitted in the interval from 4 to 20 s for alpha band and 2–20 s for beta and gamma bands^20^.

#### EEG processing

MATLAB 2020a (The MathWorks Inc., Natick, MA, 2014) was used for EEG preprocessing and analysis. Continuous EEG recordings were imported and preprocessed using EEGLAB^35^, and analyzed using custom-made scripts from a MATLAB toolbox developed at Vrije Universiteit Amsterdam (VU). All signals were visually inspected for artifacts in windows of 10 s. Noisy channels (e.g., with no or bad conductance to the scalp) and segments containing transient artifacts were removed. Next, recordings were re-referenced to the average BioSemi reference. Independent Component Analysis (ICA) was used to project signals to components that are maximally independent from each other^36,37^. Eye components were rejected. Continuous EEG recordings were segmented into conditions by pasting together all epochs of a single condition. Segmentation was done using markers of the following conditions: baseline moderate-pain stimulations (10 s each), baseline high-pain stimulations (10 s each), first eyes-closed rest (ECR1; 5 min), control acquisition stimulus (10 s each), nocebo acquisition stimulus (10 s each), control evocation stimulus (10 s each), nocebo evocation stimulus (10 s each), and second eyes-closed rest (ECR2; 5 min). Exclusion of certain segments (for example, segments that were too short for DFA computation) resulted in a varying number of participants across analyses and figures.

### Statistical analysis

EEG biomarkers were computed and tested per EEG-channel for all 32 channels. Non-parametric paired Wilcoxon signed-rank test was used to test for differences between each two conditions. Multiple-comparison corrections were performed using a False Discovery Rate procedure (FDR) with *q* = 0.05^35,36^. For the Wilcoxon signed-rank test, we reported the median of the two conditions tested, the *Z*-value and the *P*-value. To test for associations between EEG biomarkers and behavioral outcome measures, we calculated Spearman’s rank correlation coefficient (*r*_*s*_). These tests are appropriate tests for non-parametric brain-derived data and for both discrete and continuous variables, including ratio variables such as the values associated with neuronal oscillations and ordinal variables such as NRS pain ratings. On all spatial topographies, open white circles reflect statistical significance at *P* < 0.05, whereas closed white circles indicate statistical significance after FDR correction. Since some statistical effects were widespread across the cortex and others were localized above specific brain areas, we always report statistics of the whole-brain average (mean of all channels), unless stated otherwise for some instances where we additionally report statistics of specific electrodes in case of localized effects.

## Results

### Participants and pain reports

Thirty-nine participants were enrolled in this study and underwent calibration, conditioning and evocation of nocebo hyperalgesia (Fig. 1a, b). Testing of three participants was discontinued; one due to technical difficulties, one for experiencing discomfort and headache during testing, and one for not reporting differences in experienced pain between acquisition control and nocebo trials. A total of 36 participants (25 female) were included in final analyses. Mean warmth detection threshold across participants was 33.7 °C (standard deviation; SD = 0.7) and mean pain threshold was 41.9 °C (SD = 3.2). Mean temperatures used to induce moderate and high pain were 46.6 °C (SD = 0.8) and 48.1 °C (SD = 0.5), respectively. At baseline, mean NRS pain rating for control trials was 4.4 (SD = 1.7), while mean pain rating for nocebo trials was 7.4 (SD = 1.2). During nocebo acquisition, mean pain rating for control trials was 3.9 (SD = 1.8) and mean pain rating for nocebo trials was 7.3 (SD = 1.4). Following the acquisition phase of the classical conditioning paradigm, nocebo responses were observed during evocation (Fig. 1c). A repeated measures ANOVA was conducted with trial type as within-subjects factor with two levels (first evocation nocebo trial, first evocation control trial), to establish whether significant nocebo hyperalgesia was induced. There was a significant difference between NRS reports for the first nocebo and first control trial of the evocation phase (*F* (1,35) = 27.44, *p* = 0.000008, $\eta_{p}^{2}=0.44$ ) indicating the presence of nocebo hyperalgesia. There was also a significant difference across all 16 trials of the evocation phase (*F*(1,35) = 34.315, *p* = 0.000001, $\eta_{p}^{2}=0.495$ . The mean difference in NRS pain scores across all 16 evocation phase trials was 0.7 (SD = 0.8), with a mean nocebo trial score of 4.3 (SD = 2.2) and a mean control trial score of 3.6 (SD = 2.2). By the final trials of evocation, the mean difference in NRS pain scores was 0.5 (SD = 1.7), with a final mean nocebo trial score of 4.2 (SD = 2.4) and a final mean control trial score of 3.7 (SD = 2.4).

### Pre- to post-acquisition changes in LRTC are negatively associated with the magnitude of induced nocebo hyperalgesia

**Figure 2:**
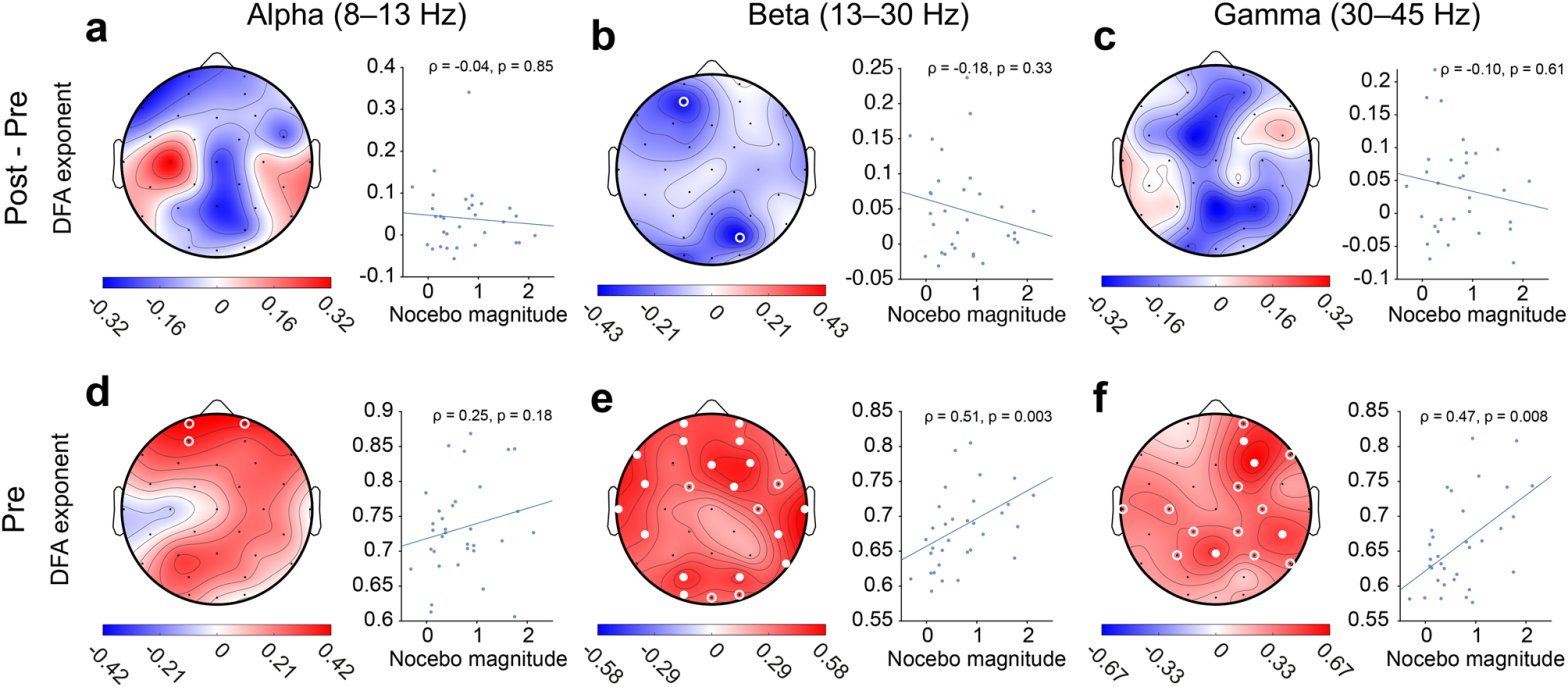
Complexity of neuronal oscillations at baseline predicts pain response to nocebo treatment. Spatial topographies show Spearman’s rank correlation coefficient values (rho) of magnitude of nocebo hyperalgesia and EEG measures (*n* = 33). Magnitude of nocebo hyperalgesia was defined as the difference between mean pain response of all nocebo trials and all control trials during the evocation phase, per individual. Top row shows the association between magnitude of nocebo hyperalgesia and EEG parameters for the difference condition ECR_POST_ − ECR_PRE_. Bottom row shows the correlation of nocebo hyperalgesia with EEG condition ECR_PRE_. (**a–c**) Magnitude of nocebo hyperalgesia was negatively associated with DFA within beta and gamma bands. (**d–f**) Individuals with high DFA beta and gamma at baseline (ECR_PRE_) show a larger nocebo effect during evocation. Red colors indicate positive correlations, whereas blue colors indicate negative correlations. Open white circles show statistical significance at *P* < .05. Closed white circles indicate significance after correcting for multiple comparisons using a False Discovery Rate procedure (FDR) with *q* = 0.05, per topography. Scatter plots show the mean correlation coefficient based on all channels.

We asked whether differences in EEG due to nocebo conditioning were associated with magnitude of nocebo hyperalgesia (Fig. 2). Our primary hypothesis was that pre- to post-acquisition differences in the alpha band would be associated with magnitudes of induced nocebo hyperalgesia. There was no significant association between change in resting-state alpha power from pre- to post-acquisition and magnitude of nocebo hyperalgesia (mean across electrodes, *r*_*s*_ =  − 0.04, *p* = 0.85). We then looked more broadly at spectral and temporal biomarkers in alpha, beta and gamma bands to test for associations with the magnitude of nocebo hyperalgesia. There were no significant differences in resting-state LRTC from pre- to post-acquisition for alpha (*r*_*s*_ =  − 0.04, *p* = 0.85), beta (*r*_*s*_ =  − 0.18, *p* = 0.33) or gamma bands (*r*_*s*_ =  − 0.10, *p* = 0.61) (Fig. 2a–c).

### LRTC of neuronal oscillations during rest predict pain response to nocebo treatment

We then asked whether resting-state EEG parameters can predict magnitude of nocebo hyperalgesia. There was no association between DFA and magnitude of nocebo hyperalgesia within the alpha band (*r*_*s*_ = 0.25, *p* = 0.18; Fig. 2d). Nocebo hyperalgesia was significantly positively correlated with whole-brain average DFA of beta (*r*_*s*_ = 0.51, *p* = 0.003) (Fig. 2e) and gamma oscillations (*r*_*s*_ = 0.47, *p* = 0.008) (Fig. 2f). These results show that individuals with strong LRTC during rest at pre-acquisition baseline have a larger nocebo effect than individuals with weak LRTC. Since stronger LRTC reflect more complex neural dynamics, these findings indicate that people with more complex brain activity are more susceptible to the acquisition of nocebo hyperalgesia.

### Nocebo conditioning suppresses power of alpha oscillations

**Figure 3:**
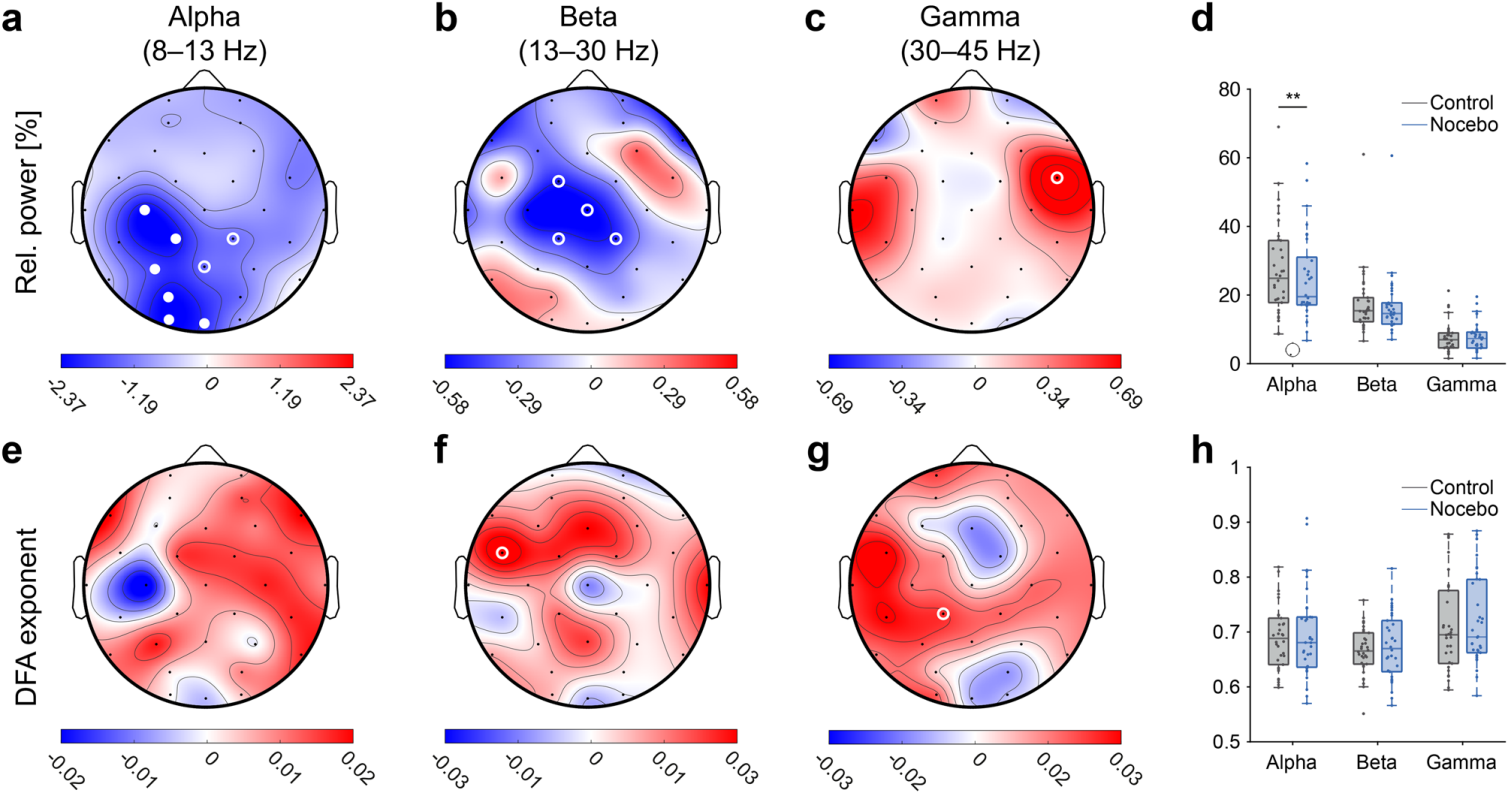
Oscillatory power of alpha oscillations is suppressed during conditioning of nocebo hyperalgesia. (**a**–**c**) Difference in relative power alpha (**a**), beta (**b**) and gamma band (**c**) for nocebo minus control trials_,_ mean of all subjects. Differences were tested for statistical significance using the non-parametric paired Wilcoxon signed-rank test. Open white circles show statistical significance at *P* < .05. Closed white circles indicate significance after correcting for multiple comparisons using a False Discovery Rate procedure (FDR) with *q* = 0.05, per topography. (**d**) Boxplots for relative power alpha, beta and gamma band. (**e**–**g**) Difference in DFA (**h**), beta (**i**) and gamma band (**j**) for nocebo minus control trials_,_ mean of all subjects. (**h**) Boxplots for DFA alpha, beta and gamma band. All boxplots show the mean of all channels, except for relative power alpha in (**g**), where the boxplots show values of electrode PO3. Electrode PO3 was chosen as a representative electrode, since the significant differences were spatially localized above parietal and occipital areas.

Next, we assessed whether parameters of resting-state EEG are altered during nocebo acquisition. To this end, non-parametric paired Wilcoxon signed-rank tests were conducted to compare differences in power and DFA between nocebo and control trials during the induction phase of the study (Table 1). Relative power of alpha oscillations was significantly lower during nocebo compared to control trials, in particular above parietal and occipital regions (Electrode PO3, *Z* = 2.73, *p* = 0.0064) (Fig. 3a). Relative power beta was not significantly different during nocebo compared with control trials (*Z* =  −0.20, *p* = 0.84) (Fig. 3b). There were no significant differences in relative power gamma between nocebo and control trials after multiple comparisons correction (*Z* =  −1.53, *p* = 0.13) (Fig. 3c, d). There were no significant differences in LRTC between nocebo and control trials within alpha (*Z* =  −0.35, *p* = 0.73), beta (*Z* =  −0.79, *p* = 0.43) and gamma bands (*Z* =  −1.43, *p* = 0.15) after multiple comparisons correction (Fig. 3e–h). We then asked whether neurophysiological changes in spectral power were also observed during the evocation phase of the study. No significant differences were observed between nocebo and control trials in the evocation phase (supplementary material).

**Table 1:** Summary of statistics for differences in EEG parameters between nocebo and control trials during conditioning shown in Fig. 2.

EEG parameter	Electrode	Mdn_CONT_	Mdn_NOC_	*Z*	*p*
Relative power alpha	WBA	17.2 ± 1.77	17.04 ± 1.58	1.75	0.08
	**PO3**	**24.88 ± 2.36**	**19.53 ± 2.12**	**2.73**	**0.0064**
Relative power beta	WBA	17.86 ± 1.13	18.55 ± 1.07	− 0.20	0.84
Relative power gamma	WBA	6.99 ± 0.75	7.33 ± 0.70	− 1.53	0.13
DFA alpha	WBA	0.69 ± 0.01	0.68 ± 0.01	− 0.35	0.73
DFA beta	WBA	0.68 ± 0.01	0.69 ± 0.01	− 0.79	0.43
DFA gamma	WBA	0.69 ± 0.01	0.69 ± 0.01	− 1.43	0.15

Wilcoxon signed-rank tests were performed on the whole-brain average per subject (computed as mean of all electrodes). Rows show EEG parameters, columns show the median whole-brain average value across subjects for control and nocebo trials, *Z*- and *P*-value corresponding to the signed-rank test. The median EEG parameter value for each group is reported with the standard error of the mean.

*CONT* Control trials during conditioning, *NOC* Nocebo trials during conditioning.

Bold font weight indicates significance at *P* < 0.05.

### LRTC and alpha power differentiate nocebo pain from high pain at baseline

**Figure 4:**
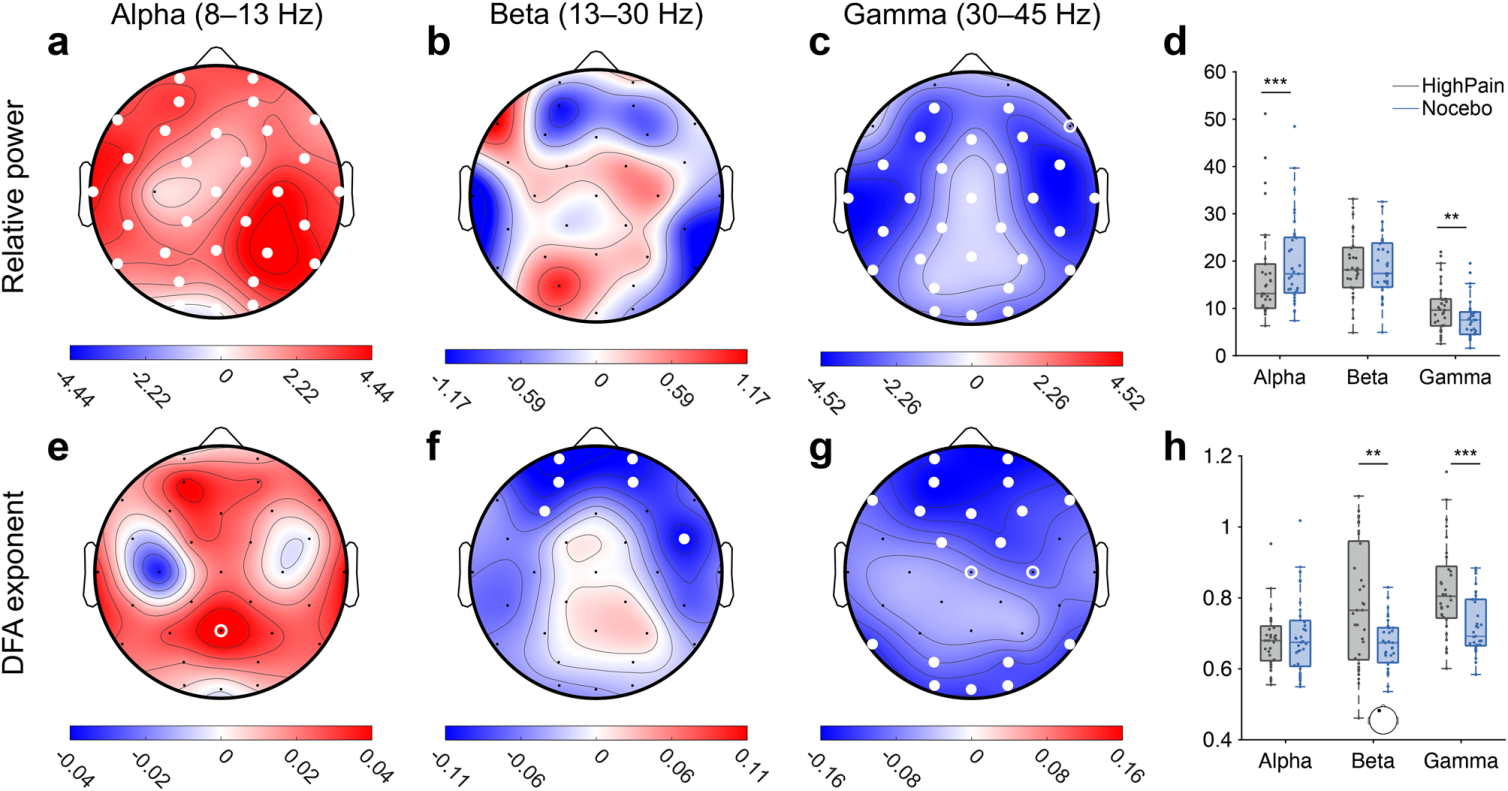
LRTC of beta and gamma oscillations differentiate nocebo pain from high pain at baseline. Spatial topographies show the mean difference NoceboEvocation—BaselineHighPain for all subjects (*n* = 33). Red colors on topographies indicate higher values for nocebo during evocation, whereas blue colors show higher values for baseline high pain. (**a–d**) Relative power of alpha oscillations was significantly higher, whereas relative power of gamma oscillations was significantly lower, during nocebo pain, compared to high pain at baseline. (**e–h**) DFA of beta and gamma oscillations—in particular above frontal regions—was significantly lower during nocebo pain compared to high pain at baseline. Differences were tested for statistical significance using the Spearman’s rank correlation coefficient. Open white circles show statistical significance at *P* < .05. Closed white circles indicate significance after correcting for multiple comparisons using a False Discovery Rate procedure (FDR) with *q* = 0.05, per topography. All boxplots show the mean across all electrodes, except for the boxplots of DFA beta in (**h**), where we show a representative electrode (AF3) of the spatially localized effect.

Our next question was whether these differences in and associations with LRTC of beta and gamma oscillations were present only during rest or if they also reflected nocebo hyperalgesia. To this end, Wilcoxon signed-rank tests were used to compare power and DFA of high pain at baseline EEG measurement with nocebo trials during the evocation phase (Fig. 4; Table 2). Compared to baseline high pain, relative power within the alpha band was significantly higher during nocebo pain (*Z* =  −3.5, *p* = 0.0004) (Fig. 4a). Relative power of gamma oscillations was lower during nocebo pain than during baseline high pain (*Z* = 3.3, *p* = 0.001) (Fig. 4c). Relative power within the beta band was not significantly different between nocebo during evocation and baseline high pain (*Z* = 0.5, *p* = 0.61) (Fig. 4b). DFA of alpha oscillations was not significantly different between nocebo during evocation and baseline high pain when looking at the whole-brain average (*Z* =  −1.31, *p* = 0.19) (Fig. 4e). DFA was lower during nocebo pain than during baseline high pain for beta (*Z* = 3.14, *p* = 0.002) and gamma band (*Z* = 3.76, *p* = 0.0002) (Fig. 4f–h). Interestingly, gamma power and LRTC were lower, suggesting that complexity of neuronal oscillations is lower, during nocebo-augmented pain compared to high pain at baseline. Indeed, based also on the results above and given the influence of higher administered pain, as can be expected, the complexity of neuronal oscillations seems to increase, from resting-state, to baseline, to nocebo-augmented pain, to high administered pain.

**Table 2:** Summary of statistics for differences in EEG parameters between nocebo during evocation and baseline high pain shown in Fig. 4.

EEG parameter	Electrode	Mdn_BHP_	Mdn_NOC_	*Z*	*p*
**Relative power alpha**	**WBA**	**13.91 ± 1.84**	**19.11 ± 1.72**	** − 3.51**	**0.0005**
Relative power beta	WBA	19.24 ± 1.19	17.83 ± 1.13	0.51	0.61
**Relative power gamma**	**WBA**	**9.37 ± 0.92**	**5.96 ± 0.79**	**3.28**	**0.001**
DFA alpha	WBA	0.68 ± 0.01	0.69 ± 0.01	− 1.31	0.19
**DFA beta**	**WBA**	**0.73 ± 0.01**	**0.68 ± 0.01**	**3.14**	**0.0017**
**DFA gamma**	**WBA**	**0.81 ± 0.02**	**0.72 ± 0.01**	**3.76**	**0.0002**

Wilcoxon signed-rank tests were performed on the whole-brain average per subject (computed as mean of all electrodes). Rows show EEG parameters, columns show the median whole-brain average value across subjects for control and nocebo trials, *Z*- and *P*-value corresponding to the signed-rank test. The median EEG parameter value for each group is reported with the standard error of the mean.

*BHP* Baseline high pain, *NOC* Nocebo trials during evocation.

Bold font weight indicates significance at *P* < 0.05.

### No significant relationship between questionnaire scores and EEG parameters

Finally, we expected that there would be a relationship between scores on pain-related questionnaires and measures of EEG. Spearman’s rank order correlations were conducted between total scores on each of the questionnaires (FPQ, PCS, and ECIP) and changes in resting-state EEG biomarker values from before to after nocebo induction. After correcting for multiple comparisons, no significant correlations were found between questionnaire scores and any of the biomarker values in any clusters of electrodes (supplementary material).

## Discussion

This study provides several new insights into the electrophysiological phenotype of nocebo hyperalgesia using EEG. Spectral and temporal dynamics of brain oscillations were studied at baseline, during resting-state pre- and post- measurements and during nocebo acquisition and evocation. The main findings of this study are (1) a positive correlation between magnitude of nocebo hyperalgesia and baseline LRTC for beta and gamma oscillations, (2) alpha power suppression during nocebo conditioning, and (3) EEG biomarker differences between the experience of high pain at baseline and the experience of nocebo-augmented pain.

Strong resting-state LRTC at baseline predicted more effective conditioning of nocebo responses. We found that, during rest, before the start of the experimental phases, strong LRTC predicted higher nocebo responses. This finding relates to the above-mentioned studies, that pointed towards an involvement of LRTC in cognitive ability^23,24,38^. Stronger LRTC reflect more complex neural dynamics and therefore, it appears that people with more complex baseline brain activity may exhibit higher cognitive functioning^24^ and are thus more susceptible to the acquisition of nocebo hyperalgesia through learning. Here, the implication of gamma band oscillations is in line with EEG research on (associative) learning, suggesting that memory encoding involves gamma oscillations^12,39,40^ potentially in coordination with hippocampal function^41^. This links gamma oscillations, which were shown to be involved in nocebo in this study, to a role of the hippocampus in learning and nocebo hyperalgesia^42,43^. It is also noteworthy that emotional processes that may play a mediating role in nocebo hyperalgesia, such as fear^44^, may engage patterns of gamma coupling in the amygdala^45^, a structure that has also been implicated in nocebo hyperalgesia^43,46,47^. Our finding of increased complexity of gamma-band oscillations in those more susceptible to nocebo hyperalgesia may thus point towards potential electrophysiological indications of specific underlying cognitive-emotional processes, such as associative learning ability as well as fear processing. Nevertheless, it should be noted that some of the results on LRTC are less robust than others and replication of these early findings in necessary for an accurate interpretation of results.

Alpha band oscillatory power has been shown to underlie the perceptual processing of incoming stimuli, including sensory perception^48^. Our study was methodologically different from the two previous studies on electrophysiological nocebo correlates^15,18^ and our results do not show consistent support of previous findings relating alpha oscillations to nocebo hyperalgesia. While our findings indicate an involvement of alpha band oscillations during acquisition, we did not find pre- to post-acquisition changes in alpha oscillations. Methodologically, it possible that the time elapsed between the first and second resting state recordings was too long, resulting in a failure to capture electrophysiological changes in alpha oscillations related to nocebo processing.

Nevertheless, we found that nocebo trials during the acquisition phase were characterized by decreased power in the alpha band, as compared to control trials. Our finding may reflect the formation of pain expectations and an inhibitory function of alpha oscillations in pain perception. Moreover, alpha-band oscillations where involved when comparing the experience of baseline high-pain stimulations to the experience of increased pain under nocebo hyperalgesic conditions, in the evocation phase. We found that there was a significant increase in alpha-band power during nocebo responses, compared to baseline pain of a matched, high intensity pain stimulus. In line with the literature, these findings may reflect the role of alpha-band oscillations in expectations^8,9^, and the cognitive regulation of pain^10,11^.

We then aimed to differentiate the temporal electrophysiological profile of experiencing high pain at baseline from that of experiencing high pain as a result of induced nocebo hyperalgesia. We found that the complexity of neuronal oscillations was lower during nocebo-augmented pain compared to baseline pain of a matched, high intensity pain stimulus. Lower oscillatory complexity during nocebo-augmented pain may be in line with our finding that lower LRTC during acquisition were associated with higher nocebo magnitudes. This could mean that the evocation of nocebo hyperalgesia, due to a state of sustained attention, may be characterized by decreased LRTC^22,49–52^. Nocebo-augmented pain seems to rely on cognitive processes such as learning, memory recall, and pain modulation. Decreased LRTC may thus indicate increased attentional load or cognitive performance during nocebo-augmented pain responses. More specifically, the decreased LRTC of gamma oscillations during nocebo evocation, as compared to the baseline high pain, may alternatively or additionally indicate a learning process. It has previously been shown that while learning new information may lead to increased gamma power or synchronization^39,41,53^, power of gamma oscillations may show a decrease after learning^54^. It is thus possible that in nocebo evocation, when learning is discontinued, gamma oscillations exhibit a decrease in power that reflects a previous active learning state. These results may thus highlight pronounced cognitive and learning-related differences between the neurophysiology of experiencing high pain and experiencing nocebo-evoked increased pain. Nevertheless, the LRTC findings in this study also highlight the intricacy of such complex biomarkers of temporal brain function and how they may characterize diverse cognitive functions and loads in different ways.

A number of limitations may have impacted the results of this study. First, aggregating trials of specific conditions into 10-s segments may have smeared out effects that could have been better captured using an event-related paradigm, in which the exact onset of each pain stimulus or response could be used to epoch the data into segments locked to each trial. Furthermore, the generalizability of our findings may be limited by the recruitment of a healthy, young participant sample. Findings of this study may not be consistent with results derived from pain patients or individuals who have experienced severe or chronic pain in the past, as their electrophysiological phenotype may differ from that of healthy people^55^. The specificity and sensitivity of these results is also unclear from a single study, and it remains to be seen whether these results could differentiate between nocebo-augmented pain and instances of comparable unpleasantness and saliency without pain, such as heightened fear or anxiety.

A number of early findings in this study can contribute towards the potential understanding of the neurophysiological phenotype of nocebo hyperalgesia. Future directions are also coming into view. It is imperative for future research to focus on the replication of results and the generalizability and translation of experimental results into clinical practice. This study highlighted novel EEG results that are related to the experimental nocebo context. EEG is a practical and relatively cost-effective method that may provide a valuable means for the identification of nocebo-augmented pain as well as nocebo contexts. For any diagnostic potentials to be realized, a next step is for future studies to replicate our findings in clinical contexts and populations, and to determine whether the findings can discriminate between pain and other unpleasant somatic symptoms such as itch or fatigue.

In sum, the present study points towards a number of novel directions regarding the electrophysiology that may underlie or mediate nocebo hyperalgesia. We identified both spectral and temporal parameters that are related to nocebo-augmented pain, with the latter presenting as the most important correlate of nocebo hyperalgesia in this study. The role of learning and attention at the electrophysiological level was highlighted through the involvement of LRTC as well as the extensive involvement of gamma oscillations under hyperalgesic conditions. These results are an important step towards identifying physiological biomarkers of nocebo hyperalgesia, a phenomenon that, to date, does not have any formal diagnostic criteria. The identification of biomarkers of nocebo hyperalgesia may thus prove imperative in the strive to identify and treat these effects.

## Supplementary Information

Supplementary Information.

## Data Availability

All supporting data will be made available to Editorial Board Members and referees at the time of submission. Materials, data, and scripts for data preprocessing and analyses will be made available via a complete publication data package on an online repository, upon publication of the study according to Leiden University policy.
