# Supplementary material for: Temporal structure of brain oscillations predicts learned nocebo responses to pain
Source: Sci Rep. 2021 May 7;11:9807. doi: 10.1038/s41598-021-89368-0 (PMC8105329; doi:10.1038/s41598-021-89368-0)
Supplement: Supplementary file 1 — Supplementary Information. [file 41598_2021_89368_MOESM1_ESM.docx]

Temporal structure of brain oscillations predicts learned nocebo responses to pain.

Mia A. Thomaidou ^# 1,2^, Joseph S. Blythe ^# 1,2^, Simon J. Houtman ^# 3^, Dieuwke S. Veldhuijzen^1,2^, Antoinette I. M. van Laarhoven^1,2,4^, Andrea W. M. Evers ^1,2,4,5^

^1^ Faculty of Social and Behavioral Sciences, Leiden University, 2333 AK Leiden, Netherlands

^2^ Leiden Institute for Brain & Cognition, 2333 ZA Leiden, Netherlands

^3^ Department of Integrative Neurophysiology, Center for Neurogenomics and Cognitive

Research, Amsterdam Neuroscience, VU University Amsterdam, 1081 HV

Amsterdam, Netherlands

^4^ Department of Psychiatry, Leiden University Medical Center, 2333 ZA Leiden, Netherlands

^5^ Medical Delta Healthy Society; Leiden University, Technical University Delft, & Erasmus University Rotterdam, Netherlands

Supplementary Information

***1. No significant neurophysiological differences between nocebo and control trials in the evocation phase***

We asked what the neurophysiological differences are between nocebo and control trials during the evocation phase of the study. To this end, non-parametric paired Wilcoxon signed-rank tests were conducted to compare differences in power, central frequency and DFA between nocebo and control trials of the evocation phase of the study.

Relative power alpha was lower above occipital regions during nocebo compared with control trials (Electrode Oz, Mdn_CONT_ = 19.91, Mdn_NOC_ = 17.37, *Z* = 2.28, *p* = 0.023, *r* = 0.4), but not significantly different for the whole-brain average (Mdn_CONT_ = 19.23, Mdn_NOC_ = 19.2, *Z* = 0.42, *p* = 0.67, *r* = 0.07). There were no differences in relative power within beta (Whole-brain average, Mdn_CONT_ = 18.04, Mdn_NOC_ = 18.06, *Z* = 1.44, *p* = 0.15, *r* = 0.25) or gamma band (Mdn_CONT_ = 6.04, Mdn_NOC_ = 6.28, *Z* = 0.33, *p* = 0.74, *r* = 0.06) between nocebo and control trials after FDR-correction. There were no differences between nocebo and control trials for central frequency within alpha (Mdn_CONT_ = 10.35, Mdn_NOC_ = 10.38, *Z* = 0.78, *p* = 0.44, *r* = 0.14) and beta (Mdn_CONT_ = 19.82, Mdn_NOC_ = 19.69, *Z* = 0.17, *p* = 0.87, *r* = 0.03). Central frequency of gamma oscillations was lower during nocebo compared with control trials (Mdn_CONT_ = 36.66, Mdn_NOC_ = 36.65, *Z* = 1.97, *p* = 0.048, *r* = 0.34) after FDR-correction. DFA within the alpha band was significantly lower during nocebo compared with control trials (Electrode C4, Mdn_CONT_ = 0.76, Mdn_NOC_ = 0.72, *Z* = 2.14, *p* = 0.033, *r* = 0.37) (Whole-brain average, Mdn_CONT_ = 0.69, Mdn_NOC_ = 0.68, *Z* = 2.35, *p* = 0.019, *r* = 0.41), although not significant after FDR-correction. DFA of beta oscillations was lower during nocebo than during control trials (Electrode P4, Mdn_CONT_ = 0.69, Mdn_NOC_ = 0.66, *Z* = 2.35, *p* = 0.019, *r* = 0.41) (Whole-brain average, Mdn_CONT_ = 0.7, Mdn_NOC_ = 0.68, *Z* = 2.03, *p* = 0.043, *r* = 0.35), but not significant after FDR-correction. There were no differences in DFA gamma between nocebo and control trials during the evocation phase of the study (Whole-brain average, Mdn_CONT_ = 0.72, Mdn_NOC_ = 0.72, *Z* = 0.15, *p* = 0.88, *r* = 0.03).

***2. No significant relationship between questionnaire scores and EEG biomarkers***

Below we list raw correlation values with corresponding p-values for all comparisons with questionnaire scores.

- **Association between FPQ and (POST-PRE)**
  - Relative power alpha: (Electrode P3, rho = -0.4, p = 0.022), (Whole brain, rho = -0.28, p = 0.114)
  - Relative power beta: (Electrode FC6, rho = 0.35, p = 0.048), (Whole brain average, rho = 0.15, 0.389)
  - Relative power gamma: (Electrode FC2, rho = 0.39, p = 0.025), (Whole brain, rho = 0.24, p = 0.171)
  - DFA alpha: (Electrode C4, rho = 0.4, p = 0.023), (Whole brain average, rho = 0.17, p = 0.331)
  - DFA beta: (Whole brain average, rho = 0.05, p = 0.763)
  - DFA gamma: (Electrode PO4, rho = 0.39, p = 0.026)
- **Association between PCS and (POST-PRE)**
  - Relative power alpha: (Whole brain average, rho = -0.15, p = 0.409)
  - Relative power beta: (Whole brain average, rho = -0.05, p = 0.778)
  - Relative power gamma: (Whole brain average, rho = 0.07, p = 0.693)
  - DFA alpha: (Whole brain average, rho = 0.13, p = 0.467)
  - DFA beta: (Whole brain average, rho = 0.12, p = 0.521)
  - DFA gamma: (Electrode FC1, rho = 0.37, p = 0.033), (Whole brain average, rho = 0.1, p = 0.595)
- **Association between ECIP and (POST-PRE)**
  - Relative power alpha: (Whole brain average, rho = -0.17, p = 0.352)
  - Relative power beta: (Electrode AF3, rho = -0.4, p = 0.021), (Whole brain, rho = -0.11, p = 0.528)
  - Relative power gamma: (Whole brain average, rho = -0.04, p = 0.818)
  - DFA alpha: (Electrode C4, rho = 0.39, p = 0.026), (Whole brain average, rho = 0.13, p = 0.468)
  - DFA beta: (Whole brain average, rho = -0.03, p = 0.853)
  - DFA gamma: (Electrode CP5, rho = -0.38, p = 0.028), (Whole brain average, rho = -0.14, p = 0.444)
